# A novel, machine-learning model for prediction of short-term ASCVD risk over 90 and 365 days

**DOI:** 10.3389/fdgth.2024.1485508

**Published:** 2024-11-01

**Authors:** Tomer Gazit, Hanan Mann, Shiri Gaber, Pavel Adamenko, Granit Pariente, Liron Volsky, Amir Dolev, Helena Lyson, Eyal Zimlichman, Jay A. Pandit, Edo Paz

**Affiliations:** ^1^ Hello Heart, Inc., Menlo Park, CA, United States; ^2^ Sheba Medical Center, Tel Hashomer, Israel; ^3^ Scripps Research Translational Institute, La Jolla, CA, United States

**Keywords:** machine learning, cardiovascular risk assessment, personalized preventive strategies, mobile health, digital health technology

## Abstract

**Background:**

Current atherosclerotic cardiovascular disease (ASCVD) risk assessment tools like the Pooled Cohort Equations (PCEs) and PREVENT™ scores offer long-term predictions but may not effectively drive behavior change. Short-term risk predictions using mobile health (mHealth) data and electronic health records (EHRs) could enhance clinical decision-making and patient engagement. The aim of this study was to develop a short-term ASCVD risk prediction model for hypertensive individuals using mHealth and EHR data and compare its performance to existing risk assessment tools.

**Methods:**

This is a retrospective cohort study including 51,127 hypertensive participants aged ≥18 years old who enrolled in the Hello Heart CV risk self-management program between January 2015 and January 2024. A machine learning (ML) model was derived from EHR data and mHealth measurements of blood pressure (BP) and heart rate (HR) collected via at-home BP monitors. Its performance was compared to that of PCE and PREVENT.

**Results:**

The XgBoost model incorporating 291 features outperformed the PCE and PREVENT scores in discriminating ASCVD risk for both prediction periods. For 90-day prediction, mean C-statistics were 0.81 (XgBoost) vs. 0.74 (PCE) and 0.65 (PREVENT). Similar findings were observed for 365-day prediction. mHealth measurements incrementally enhanced 365-day risk prediction (ROC-AUC 0.82 vs. 0.80 without mHealth).

**Conclusion:**

An EHR and mHealth-based ML model offers superior short-term ASCVD prediction compared to traditional tools. This approach supports personalized preventive strategies, particularly for populations with incomplete features for PCE or PREVENT. Further research should explore this novel risk prediction framework, and particularly additional mHealth data integration for broader applicability and increased predictive power.

## Introduction

The current approach to primary prevention of atherosclerotic cardiovascular disease (ASCVD) includes risk assessment using tools like the American College of Cardiology/American Heart Association Pooled Cohort Equations (PCEs) which are integrated into guidelines for blood pressure (BP) and cholesterol management (1, 2). Recently, the American Heart Association (AHA) developed the Predicting Risk of cardiovascular disease EVENTs (PREVENT™) equations for ASCVD and heart failure (HF) risk prediction (3). Both the PCEs and PREVENT estimate risk over 10 years, 30 years, or an individual's lifetime (1, 4). However, research suggests that communicating shorter-term ASCVD risk may better motivate patients to adopt healthier behaviors and adhere to treatment, potentially enhancing preventive efforts (5).

Electronic health records (EHRs) provide comprehensive health data, enabling the creation of risk assessment algorithms for a wide range of conditions. Recently, EHR-based machine learning (ML) models have been developed for predicting coronary artery disease (CAD), myocardial infarction (MI) and stroke (6, 7). EHR data was found to improve CAD prediction by approximately 10% compared to the PCEs (8).

Given that more than half of adults with hypertension monitor their BP at home (9), and 90% of US adults now own smartphones (10), mobile health (mHealth) can provide an additional data source for refinement of ASCVD risk prediction. These technologies enable the collection of health metrics such as heart rate (HR), and BP at home, bridging the gap between sporadic clinical visits.

In this study, we aimed to develop a short-term ASCVD ML prediction model for a hypertensive population based on EHR and mHealth data. The mHealth data was obtained using Hello Heart, an mHealth cardiovascular (CV) risk self-management program that consists of a Bluetooth-enabled BP monitor and connected smartphone application (app). We investigated two key research questions: (1) What is the enhancement in short-term ASCVD risk prediction offered by this ML model compared to PCE and PREVENT? and (2) Can this model broaden the applicability of risk assessment to populations lacking the complete set of requisite features for PCE and PREVENT?

## Methods

### Sample population

This is a retrospective cohort study of participants ≥18 years of age who enrolled in the Hello Heart program between January 2015 and January 2024 in the US. Individuals with confirmed hypertension through prior diagnosis or relevant medical/pharmacy insurance claims were eligible to enroll voluntarily. All participants consented to terms permitting research use of de-identified, encrypted data. Only those who connected their EHRs to the app and had at least one EHR record were included in the analysis. The program is Health Insurance Portability and Accountability Act (HIPAA) compliant. This study was reviewed by the Western Institutional Review Board-Copernicus Group (WCG) Institutional Review Board (IRB tracking ID 20226635) and determined to be exempt under 45 CFR 46.104(d)(4). In addition, a waiver of HIPAA authorization for the use and disclosure of aggregated, de-identified data was obtained. No compensation was provided to participants.

### ASCVD classification

To define dates for ASCVD events, including acute coronary syndrome (ACS) or cerebrovascular accident (CVA), we developed a semi-automatic process. Participants with ACS or CVA identified using International Classification of Diseases (ICD)-9, ICD-10 or SNOMEDCT codes (Supplementary Tables S1, S2) and with a specific date of onset were considered positive cases with the specified dates. For those without a specific date of onset, trained medical annotators (LV & GP) completed manual chart review and assigned a date of onset according to the guidelines described in the Supplementary Appendix. Cases lacking validated dates were excluded, and only the first validated event per participant was used. Supplementary Figures S1, S2 detail the ASCVD event classification and number of participants and events.

### Data sources and features

Seven EHR resource type tables, stored with HL7 FHIR specification, were used to extract features relevant for the model: participant, condition, encounter, family history, medication statement, observations, and procedures. In addition, BP (including systolic and diastolic BP) and HR were collected through the mHealth app and an accompanying FDA-cleared home BP monitor (Zewa UAM-910BT, Zewa UAM-900 T, or A&D UA-651BLE BP cuffs). We considered both categorical and continuous data as clinical features. For categorical data, presence of a diagnostic code/medication prescription in the EHR was coded as “1”; absence was coded as “0”. In total, 291 features were derived (Supplementary Table S3): 9 demographics, 182 observations (including BP, HR, and cholesterol), 10 conditions, 3 medications, 19 procedures, 9 family history and 3 home monitoring data were used in the analyses. Age bins of 18–50, 50–70 and >70 were also added to the model (11). Three features were added for (1) the number of records prior to the selected date, (2) the number of EHR records in the 90 days before the selected date, and (3) the number of mHealth measurements in the 90 days before the selected date. Systolic BP, diastolic BP and HR were obtained from both the EHR and home monitoring devices.

Neural network models allow the summarization of participants’ medical status in the form of embedding vectors (12). Finch et al. (13) used the Word-2-Vec model (14) to create medical concept embeddings for ICD-10 codes. We utilized these embeddings to summarize each participant's medical condition at a given time into a 50-value vector. These values were added to the previously described features. Missing values were imputed using mean imputation for continuous variables.

### PCE and PREVENT calculations

The PCE and the PREVENT scores were calculated using available formulas (15, 16). For the PCE, we applied the non-Hispanic White equations. Race data was missing for most cases, but among those where it was available, the majority were White. Additionally, PCE estimates do not improve with the addition of race (17), and AHA guidelines suggest using the non-Hispanic White equations for populations other than African Americans and non-Hispanic Whites (18). Race was not required for the PREVENT score. The PREVENT score was calculated using the basic 10-year model for ASCVD version which includes eGFR but not Hemoglobin A1C, Social Deprivation Index (SDI) or albumin-to-creatinine ratio (ACR). Requiring these additional features would significantly reduce eligible participants and was reported to yield limited performance improvement (15). Scores were calculated only for participants that had all required features.

### ML model

To determine ML risk prediction performance, the study population was split into a 70% training set and a 30% test set stratified by outcome. An Ensemble Boosting Tree-based model (XgBoost), was chosen for its ability to model complex, high-order interactions between the input variables. Hyperparameter tuning was performed using stratified cross validation of the training set and sequential grid search. Model calibration was performed using cross-validation classifier calibration (19). Analysis was performed in Python 3.10 using the Scikit-learn and XgBoost packages (20), Versions 1.3.0 and 1.7.5, respectively.

Two prediction periods were evaluated (90 and 365 days), and five models were evaluated for each prediction period and compared to the PCE and PREVENT calculators:

(1) *All features*: XgBoost model applied to all users that meet initial criteria and modeled with all available features.
(2) *All features-PCE eligible*: XgBoost model applied only on users that have the features required for the PCE (but using all features).
(3) *PCE features*: XgBoost model applied only on users that have the features required for the PCE and using only features required for the PCE.
(4) *All features-PREVENT eligible*: XgBoost model applied only on users that have the features required for PREVENT (but using all features).
(5) *PREVENT features:* XgBoost model applied only on users that have the features required for PREVENT and using only features required for PREVENT.

A “prediction date” was set for each participant, marking the last date data could be included for modeling. Data beyond this date was excluded. For positive cases (those with ASCVD events), a random date up to 90 or 365 days before the event was chosen. Negative cases (those without ASCVD events) had a random date selected after their first EHR entry. Each resource type had a designated lookback period (time before the prediction date on which data was accumulated) and aggregation methodology. Lookback period was not limited for family history, smoking, alcohol, medications, conditions procedures and embeddings. Lookback period was limited to 10 years for observations (including BP and cholesterol). For example, observation data points were monitored for 10 years prior to the prediction date and each observation feature was aggregated using exponential weighted average. Supplementary Table S4 shows the different lookback periods and aggregation protocols.

**Figure 1:**
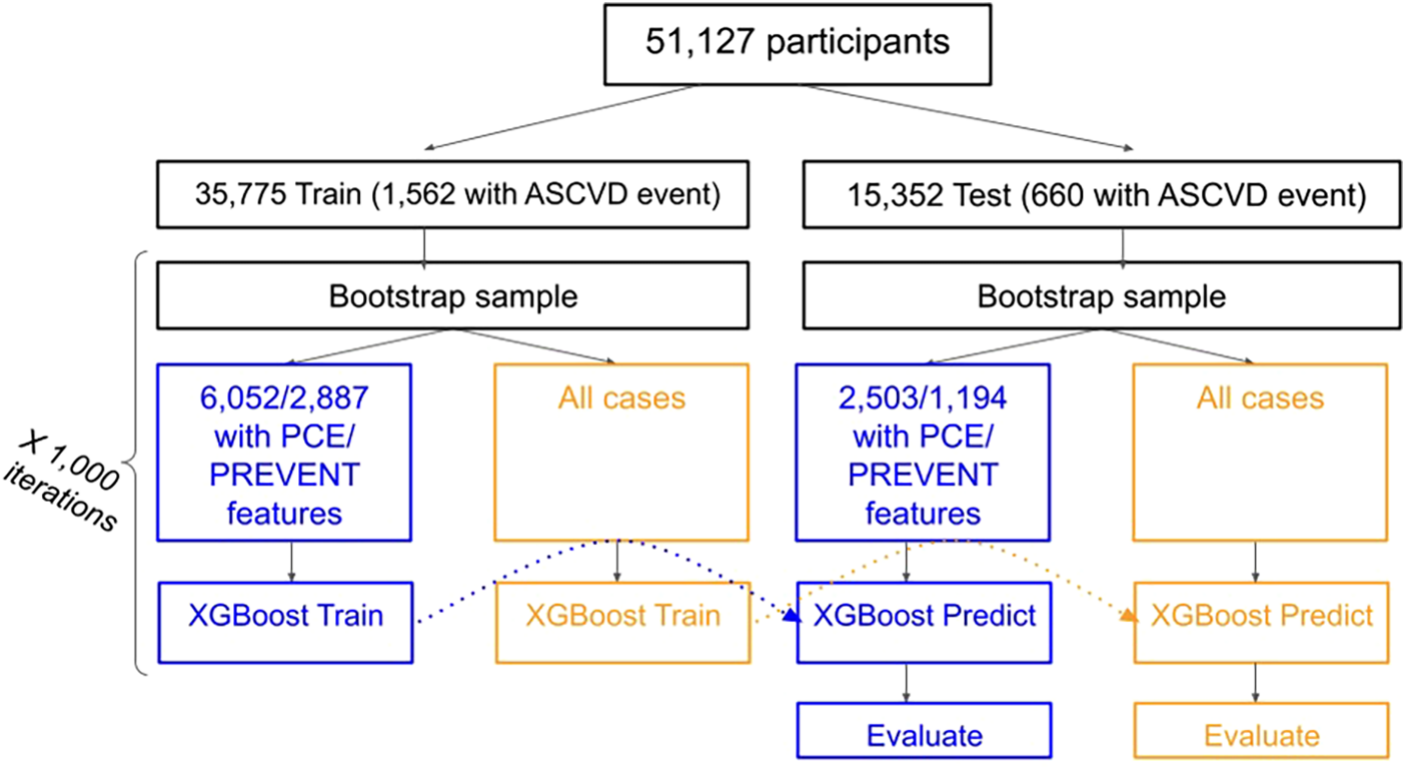
Study design and flowchart.

PCE and PREVENT scores were derived using the relevant aggregated features. Study design and flowchart is shown in Figure 1.

### Validation and statistics

We evaluated the performance of the models using C-statistic (ROC-AUC) and compared the difference in C-statistic between the models. Both train and test sets were sampled 1,000 times using bootstrapping sampling. 1,000 models were trained on the sampled train set and evaluated on the sampled test set. Reported performance metrics are the mean value and Confidence Intervals (CIs) across all predictions performed with each model. An additional bootstrapping-based evaluation was performed to examine the additive value of mHealth BP and HR measurements over a baseline of EHR data. Additional information regarding sampling, bootstrapping, and model evaluation can be found in the Supplementary Appendix.

## Results

### Study population and characteristics

Of 54,867 participants, 51,227 matched the inclusion criteria. 51,127 (35,775 train and 15,352 test) remained after excluding cases who had an ICD code for ASCVD but the date could not be verified. Demographic characteristics of the population are described in Table 1. 2,222 cardiovascular events (for 2,222 participants) were found: 1,562 (721 ACS, 841 CVA) and 660 (329 ACS, 331 CVA) in the train and test sets respectively. 8,555 participants had all PCE features (excluding race): 6,052 in the training set (406 with event) and 2,503 in the test set (170 with events). 4,081 participants had all PREVENT features: 2,887 in the train set (218 with event) and 1,194 in the test set (92 with events).

**Table 1:** Population demographics and characteristics.

Characteristic		Population group			
		Overall	Test	Train	Missing
*n*		51,127	15,352	35,775	
label, *n* (%)^a^	0 (no events)	48,905 (95.7)	14,692 (95.7)	34,213 (95.6)	0
label, *n* (%)^a^	1 (event)	2,222 (4.3)	660 (4.3)	1,562 (4.4)	
age, median [Q1,Q3]^a,b^		49.0 [39.0, 58.0]	49.0 [39.0, 58.0]	49.0 [39.0, 58.0]	1,691
Sex, *n* (%)^a,b^	Female	20,658 (40.4)	6153 (40.1)	14,505 (40.5)	0
Sex, *n* (%)^a,b^	Male	17,169 (33.6)	5205 (33.9)	11,964 (33.4)	
Sex, *n* (%)^a,b^	Unknown	13,300 (26.0)	3,994 (26.0)	9,306 (26.0)	
Diabetes type I, *n* (%)^a^	Yes	291 (0.6)	97 (0.6)	194 (0.5)	
Diabetes type II, *n* (%)^a^	Yes	4,377 (8.6)	1,303 (8.5)	3,074 (8.6)	
High cholesterol, *n* (%)^b^	Yes	20,514 (40.1)	6,158 (40.1)	14,356 (40.1)	
Anxiety, *n* (%)^b^	Yes	14,376 (28.1)	4,345 (28.3)	10,031 (28.0)	
Depression, *n* (%)^b^	Yes	8,737 (17.1)	2,666 (17.4)	6,071 (17.0)	
Smoking (ever), *n* (%)^a,b^	Yes	17,339 (33.9)	5,210 (33.9)	12,129 (33.9)	
Region of country, *n* (%)^b^	Midwest	9,784 (19.1)	2,895 (18.9)	6,889 (19.3)	0
Region of country, *n* (%)^b^	Northeast	5,673 (11.1)	1,722 (11.2)	3,951 (11.0)	
Region of country, *n* (%)^b^	South	28,265 (55.3)	8,468 (55.2)	19,797 (55.3)	
Region of country, *n* (%)^b^	West	7,275 (14.2)	2,232 (14.5)	5,043 (14.1)	
Region of country, *n* (%)^b^	Unknown	130 (0.3)	35 (0.2)	95 (0.3)	
Systolic BP, median [Q1, Q3]^a,b^		129.8 [129.8, 129.8]	129.8 [129.8, 129.8]	129.8 [129.8, 129.8]	0
Diastolic BP, median [Q1, Q3]^a,b^		80.2 [80.2, 80.2]	80.2 [80.2, 80.2]	80.2 [80.2, 80.2]	0

^a^ EHR.

^b^ App.

### ML model and PCE performance

**Figure 2:**
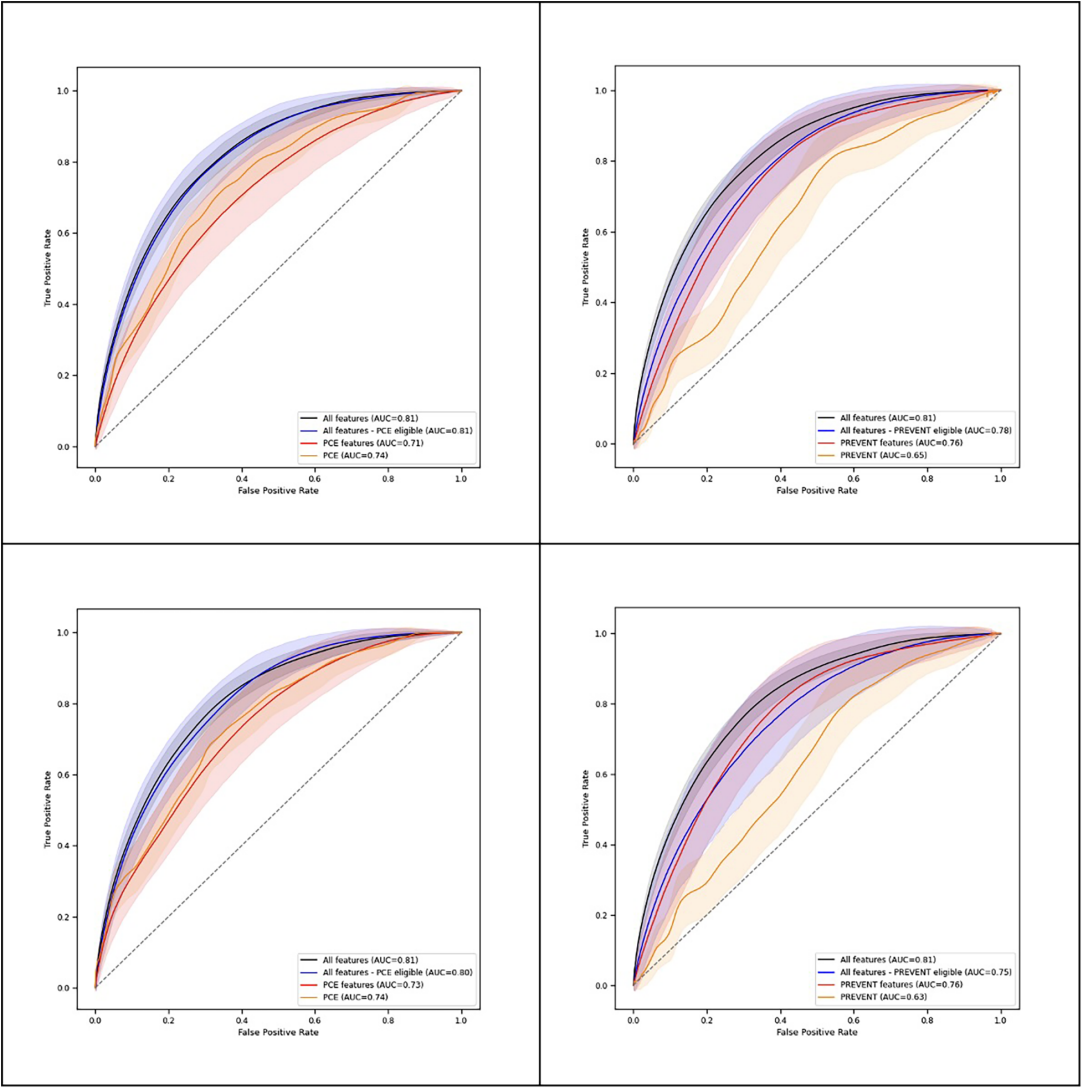
ROC plots for different models - 90 days (top), 365 days (bottom).

Table 2 shows C-statistics for the different models across 90- and 365-day prediction periods, while Figure 2 presents corresponding ROC curves. In both prediction periods, C-statistic was higher for the model derived with all features for PCE eligible participants compared to the PCE score. C-statistics was also higher for the model derived with all features for the PREVENT eligible participants compared to the PREVENT score (Table 3). Net reclassification index (NRI) and integrated discrimination index (IDI) also show an improved classification for the described models over the PCE and PREVENT models. Supplementary Figure S3 shows the positive rate for high model predictions (4th quantile) vs. positive rate for low model predictions (1st quantile).

**Table 2:** Performance for 90- and 365-day prediction periods.

Model	C-statistics (90 days)	C-statistics (365 days)
All features	0.81 [0.8–0.83]	0.81 [0.79–0.82]
All features - PCE eligible	0.81 [0.78–0.84]	0.8 [0.77–0.83]
PCE features	0.71 [0.67–0.75]	0.73 [0.69–0.77]
PCE	0.74 [0.71–0.78]	0.74 [0.71–0.78]
All features - PREVENT eligible	0.78 [0.73–0.82]	0.75 [0.69–0.81]
PREVENT features	0.76 [0.71–0.8]	0.76 [0.7–0.81]
PREVENT	0.65 [0.6–0.7]	0.63 [0.57–0.68]

**Table 3:** Model comparison - 90 days and 365 days.

Comparison	dAUC (90 days)	NRI (90 days)	IDI (90 days)	dAUC (365 days)	NRI (365 days)	IDI (365 days)
All features-PCE eligible vs. PCE	0.07 [0.03–0.1]*	0.2 [0.0–0.37]*	0.05 [0.01–0.09]*	0.06 [0.02–0.09]*	0.17 [−0.03–0.38]	0.03 [−0.01–0.07]
All features-PCE eligible vs. PCE features	0.1 [0.06–0.14]*	0.65 [0.48–0.84]*	0.09 [0.04–0.14]*	0.07 [0.03–0.11]*	0.59 [0.39–0.77]*	0.06 [0.01–0.1]*
All features-PREVENT eligible vs. PREVENT	0.13 [0.07–0.19]*	0.37 [0.11–0.64]*	0.08 [0.02–0.16]*	0.13 [0.06–0.19]*	0.3 [0.01–0.59]*	0.08 [0.01–0.17]*
All features-PREVENT eligible vs. PREVENT features	0.02 [−0.03–0.07]	0.11 [−0.39–0.54]	0.03 [−0.05–0.12]	0.0 [−0.06–0.05]	−0.39 [−0.68 to −0.07]*	0.01 [−0.07–0.1]

*p < 0.05.

### Feature importance

Multiple features contribute to the predictive capabilities at both 90 and 365 days. Supplementary Figure S4 shows Shapley values for the all-feature model. Top features included cholesterol and BP medication, age, number of records and family history of CAD.

### Additive value of mHealth BP and HR measurements

We restricted our analysis to 1,059 participants who had home measurements of BP and HR before the prediction date to evaluate the contribution of mHealth data. These included 56 participants with ASCVD events (32 ACS, 24 CVA). In this group, we compared a model with all features to a model with systolic BP, diastolic BP and HR from the home monitor removed. BP and HR measurements were still available from the EHR in both models. For 365-day prediction, we found a slightly higher ROC-AUC for the model that included mHealth BP and HR measurements [0.82 (0.76–0.86) vs. 0.80 (0.73–0.86), dAUC 0.01 (−0.01–0.04), NRI 0.11 (−0.38–0.57), IDI 0.01 (−0.03–0.04)]. For the 90-day prediction period, similar ROC-AUC values (Supplementary Figure S5) were observed for the model with [0.85 (0.81–0.90)] and without [0.85 (0.82–0.89)] mHealth measures [dAUC 0.00 (−0.02–0.02), NRI 0.13 (−0.53–0.60), IDI [−0.02 (−0.07–0.03)]. Shapley values showed mHealth-measured systolic BP as a top feature for both 90- and 365-day prediction (Supplementary Figure S6).

## Discussion

A key step in ASCVD treatment and prevention is estimating individual risk (1). However, traditional risk calculators provide long-term risk estimates that can be difficult for individuals to internalize, and fear or unwillingness to begin drug therapy or adhere to healthy lifestyle recommendations may outweigh perceived risk of heart disease (21). In this study, we developed an mHealth and EHR-based ML model that can predict the occurrence of ASCVD events (including ACS and CVA) within a short time frame of 90 or 365 days in a population of hypertensive participants. Communicating cardiovascular risk to individuals in a shorter time frame may improve their understanding of risk and better incentivize risk reduction and behavior change (5).

Our short-term risk prediction model's performance compared favorably with the PCEs and PREVENT. For a set of participants with all the features required to complete PCE risk estimation, we found that an XgBoost model with 291 features provided improved discrimination compared to the PCE score and to a model based on PCE features for both 90- and 365-day prediction. For a set of participants with all the features required to complete PREVENT risk estimation, the model improved discrimination compared to the PREVENT score for both 90- and 365-day prediction and to a model based on PREVENT features for 90-day prediction.

PCEs and PREVENT, moreover, use a small number of traditional risk factors, which, though highly predictive of ASCVD events, offer a narrow view of an individual's health. By contrast, EHR data provides a comprehensive medical profile, revealing additional features our model found to improve classification. High serum levels of alkaline phosphatase, for example, are not considered in current risk prediction tools but have been linked to increased ASCVD risk (22). In addition, novel ML techniques allow vast amounts of clinical data to be condensed in the form of representative embeddings. We supplemented the more classical EHR features with such an approach, transforming the list of condition codes into an embedding vector (12) and feeding this to the XgBoost model. Notably, the model found some of the embeddings significant for enhancing prediction.

As anticipated, some of the most significant factors influencing the prediction, as revealed by the SHAP (SHapley Additive exPlanations) value analysis, were well-established risk factors also considered in the longer-term risk scores. These include age, gender (with males being at higher risk), smoking and the use of prescription blood pressure and cholesterol medications (higher risk for users taking medications). This study demonstrates that these factors are also relevant for predicting short term risk of 365 and even 90 days. Interestingly, total, LDL and HDL cholesterol were found to be less important. These factors did not appear among the most significant features (Supplementary Figure S4) except for HDL, which ranked as the 15th most important feature for the 365-day prediction. The impact of elevated cholesterol on ASCVD risk is known to be long term, potentially explaining its reduced significance in short term predictions (23). It is also likely that high cholesterol was indirectly captured through its correlation with another top feature, cholesterol medication use. Therefore, this conclusion should be interpreted with caution and warrants further investigation in future studies, ideally using causal models or prospective study designs. Additionally, family history of stroke and CAD emerged among the top 20 features, suggesting that these factors should be given careful consideration by clinicians, even if they are not included in standard risk scores (24).

An intriguing, aggregated feature that counted the total number of records in the EHR appeared among the top ten features. Patients with a larger volume of medical records prior to the event tended to show an elevated ASCVD risk, likely indicating a poorer health history and condition control. Another feature considered was the number of records within a more recent time frame (past 90 days). This feature ranked highly for predicting events within a 90-day window but not for the longer 365-day prediction period. Additionally, a few notable observations emerged, such as the importance of elevated serum glucose and alkaline phosphatase levels, corroborating previous research and underscoring their clinical relevance (22, 25).

While comprehensive, EHR data is only generated by interactions with an individual's healthcare providers or ancillary services. mHealth offers the opportunity to complement these data sources with higher measurement cadence. Furthermore, home BP monitoring is recommended for all hypertensive individuals to help assess treatment effectiveness (26). A trend for improved classification for the model which includes mHealth measurements was observed for the 365-day but not 90-day prediction period. Though results were from a small subset of the population with mHealth measurements prior to the ASCVD event (1,059 cases), they demonstrate the potential additive predictive power of mHealth alongside EHR data. Recognizing the promising benefits of incorporating mHealth data, we see the need for strategies to enhance adoption of mHealth home monitoring tools among broad segments of the population and downstream use of this data by providers. These strategies can include increased education about potential benefits, streamlined workflows to incorporate data into provider workflows/EHRs, improved reimbursement for providers who leverage such data, and targeted outreach to diverse populations. Studies have demonstrated that mHealth programs can close healthy equity gaps (27) and improve clinical outcomes (28). Such strategies will allow increased adoption of mHealth technologies, thus incorporating additional home monitoring biosignals within larger databases. This adoption will in turn advance, expand, and validate this study's approach for combining EHR data with mHealth signals in the context of risk prediction.

Finally, an additional drawback of PCE and PREVENT is their need for all relevant factors to calculate the risk score. Since boosting trees can manage missing data, this requirement is not necessary for calculating predictions. In our dataset, only 8,555 of 46,590 participants (18%) had all features required for the PCE. Similarly, only 3,965 users had all required features for PREVENT. As such, enabling predictions for the entire population without compromising performance is another advantage of the proposed risk model. PREVENT models with additional predictors, namely ACR, A1C and SDI were also considered. However, as these additions only yielded minimal discriminative enhancement and would necessitate further reduction of the study population, we opted to compare our findings to the base model offered for ASCVD.

Overall, these findings suggest that an EHR and mHealth-based ML model can predict ASCVD within a short time frame, with higher discrimination, and for a broader population than the other models evaluated.

## Limitations

Our study has several limitations. First, our dataset consists of participants with high BP enrolled in a mHealth CV self-management program, limiting generalizability. Although our population was slightly younger (mean age 49 ± 13), variables like diabetes prevalence (9.3%), and average systolic BP (129 ± 15.7) were comparable to PCE (age 52 ± 9.6, diabetes 10.3%, systolic BP 125 ± 18.7) and PREVENT (age 57 ± 12, diabetes 11.4%, systolic BP 125 ± 15.6) data. While hypertension is a prevalent condition, affecting the majority of the adult population over 40 years old (29), we recognize that our study's focus on hypertensive individuals enrolled in an mHealth program limits the generalizability of the findings. Further validation across diverse populations is warranted to ensure applicability beyond those evaluated here. While PCE and PREVENT models aren't optimized for short-term prediction, we also compared a simpler model based only on required PCE or PREVENT features but optimized for 90- and 365-day predictions. Notably, the more complex model demonstrated higher discrimination performance in the case of the PCE score.

The susceptibility of EHRs to noise, errors, and missing data, stemming primarily from data collection processes has been previously reported (30). To address these challenges in ASCVD event tagging, we implemented a semi-automatic approach in which the majority of cases were labeled automatically (using ICD and SNOMED codes) and the remaining ambiguous cases were manually annotated by experienced medical annotators, focusing on unstructured EHR data. While manual chart review can introduce subjectivity and annotator inconsistency, we anticipate that this will not significantly affect the overall outcome. Firstly, only 163 of the 1,418 potential ACS cases and 96 of the 1,528 potential CVA cases required manual intervention. Secondly, annotators were provided with strict guidelines to minimize subjectivity. These guidelines required specific conditions, such as cardiovascular-related hospital stays exceeding 12 hours and targeted medical tests performed during those hospitalizations, to classify cases as ACS or CVA. Thirdly, the obtained ACS and CVA prevalences are aligned with reported prevalences in the literature. Finally, the same tagged events were used to analyze our novel ML model and the comparator models, further mitigating the potential impact on the relative performance. Tagging errors are still possible in both the automatic and manual processes, but we believe that the balance between automated processes and manual intervention helps mitigate the risk of label noise while still accounting for edge cases (Supplementary Figures S1, S2).

## Conclusion

An ML model based on EHR and mHealth data demonstrates superior discriminative power as compared to PCE or PREVENT in predicting short-term ASCVD occurrence among a hypertensive population. Preliminary results from a smaller sub-group also indicate a trend for an additive predictive value of self-measured, mHealth-derived BP and HR measurements when combined with EHR data. The model is also applicable to a broader population, including individuals who do not have all features required for PCE or PREVENT. This model may serve as a potential tool to predict short-term risk of ASCVD in a hypertensive population, and can therefore assist in clinical decision-making, encourage behavioral and lifestyle changes, and prompt further clinical evaluations.

## Data Availability

The datasets presented in this article are not readily available because in accordance with privacy and commercial use agreements, the data sets generated during and/or analyzed in the present study are not publicly available. Requests to access the datasets should be directed to Tomer Gazit, tomer.gazit@helloheart.com.
