# Supplementary material for: A novel, machine-learning model for prediction of short-term ASCVD risk over 90 and 365 days
Source: Front Digit Health. 2024 Nov 1;6:1485508. doi: 10.3389/fdgth.2024.1485508 (PMC11564171; doi:10.3389/fdgth.2024.1485508)

Supplementary Material

1 Supplementary Text

ASCVD Event Classification

ASCVD classification initiated with a search for ASCVD (ACS or CVA) condition, as depicted by ICD9, ICD10 and SNOMEDCT codes (Table S1-S2). Following, a semi-automated labeling algorithm was applied (Figures S1-S2). Participants were classified to three subgroups:

1. Participants with a specific documented date of onset were considered positive cases with the specified dates.
2. Participants with the ASCVD codes but not in subgroup 1 due to no specific date were evaluated manually by trained medical annotators (LV & GP) according to the guidelines described below. Verified cases were considered positive examples. Non-verified cases were excluded from the analysis.
3. Participants not in subgroups 1 or 2 but with a code for a historical ASCVD event were excluded because their date could not be verified.

For participants with more than one validated event, only the first event was used.

Guidelines for Manual Labeling of Subgroup 2

ACS

1. Unstructured EHR data was manually searched for admission of more than 12 hours with clear ACS cause.
2. For cases with unstructured admission, annotators were instructed to search for ECG or Troponin during the admission. Cases that matched these conditions were considered validated cases from unstructured data.
3. Non-validated cases were excluded from the analysis.

CVA

1. Unstructured HER data were manually searched for admission of more than 12 hours with clear CVA cause.
2. For cases with unstructured admission, annotators were instructed to search for Head CT or CTA during the admission. Cases that matched these conditions were considered validated cases from unstructured data.
3. Non-validated cases were excluded from the analysis.

2 Supplementary Tables and Figures

2.1 Supplementary Tables

Supplementary Table 1. Medical codes used for ACS and CVA event classification.

Name	System	Code
ACS	ICD10CM	I20.0
ACS	ICD10CM	I21
ACS	ICD10CM	I21.0
ACS	ICD10CM	I21.01
ACS	ICD10CM	I21.02
ACS	ICD10CM	I21.09
ACS	ICD10CM	I21.1
ACS	ICD10CM	I21.11
ACS	ICD10CM	I21.19
ACS	ICD10CM	I21.2
ACS	ICD10CM	I21.21
ACS	ICD10CM	I21.29
ACS	ICD10CM	I21.3
ACS	ICD10CM	I21.4
ACS	ICD10CM	I21.9
ACS	ICD10CM	I21.A
ACS	ICD10CM	I21.A1
ACS	ICD10CM	I21.A9
ACS	ICD10CM	I22
ACS	ICD10CM	I22.0
ACS	ICD10CM	I22.1
ACS	ICD10CM	I22.2
ACS	ICD10CM	I22.8
ACS	ICD10CM	I22.9
ACS	ICD10CM	I24
ACS	ICD10CM	I24.0
ACS	ICD10CM	I24.1

ACS	ICD10CM	I24.8
ACS	ICD10CM	I24.9
ACS	ICD10CM	I25.110
ACS	ICD10CM	I25.700
ACS	ICD10CM	I25.710
ACS	ICD10CM	I25.720
ACS	ICD10CM	I25.730
ACS	ICD10CM	I25.750
ACS	ICD10CM	I25.760
ACS	ICD10CM	I25.790
ACS	ICD9CM	410.0
ACS	ICD9CM	410.00
ACS	ICD9CM	410.01
ACS	ICD9CM	410.02
ACS	ICD9CM	410.1
ACS	ICD9CM	410.10
ACS	ICD9CM	410.11
ACS	ICD9CM	410.12
ACS	ICD9CM	410.2
ACS	ICD9CM	410.20
ACS	ICD9CM	410.21
ACS	ICD9CM	410.22
ACS	ICD9CM	410.3
ACS	ICD9CM	410.30
ACS	ICD9CM	410.31
ACS	ICD9CM	410.32
ACS	ICD9CM	410.4
ACS	ICD9CM	410.40
ACS	ICD9CM	410.41

ACS	ICD9CM	410.42
ACS	ICD9CM	410.5
ACS	ICD9CM	410.50
ACS	ICD9CM	410.51
ACS	ICD9CM	410.52
ACS	ICD9CM	410.6
ACS	ICD9CM	410.60
ACS	ICD9CM	410.61
ACS	ICD9CM	410.62
ACS	ICD9CM	410.7
ACS	ICD9CM	410.70
ACS	ICD9CM	410.71
ACS	ICD9CM	410.72
ACS	ICD9CM	410.8
ACS	ICD9CM	410.80
ACS	ICD9CM	410.81
ACS	ICD9CM	410.82
ACS	ICD9CM	410.9
ACS	ICD9CM	410.90
ACS	ICD9CM	410.91
ACS	ICD9CM	410.92
ACS	ICD9CM	411.1
ACS	ICD9CM	411.8
ACS	ICD9CM	411.81
ACS	ICD9CM	411.89
ACS	SNOMEDCT_US	12238111000119106
ACS	SNOMEDCT_US	12238151000119107
ACS	SNOMEDCT_US	142025003
ACS	SNOMEDCT_US	155304006

ACS	SNOMEDCT_US	155308009
ACS	SNOMEDCT_US	15712841000119100
ACS	SNOMEDCT_US	15712881000119105
ACS	SNOMEDCT_US	15712921000119103
ACS	SNOMEDCT_US	15712961000119108
ACS	SNOMEDCT_US	15713041000119103
ACS	SNOMEDCT_US	15713081000119108
ACS	SNOMEDCT_US	15713121000119105
ACS	SNOMEDCT_US	15713161000119100
ACS	SNOMEDCT_US	15713201000119105
ACS	SNOMEDCT_US	15960061000119102
ACS	SNOMEDCT_US	15960541000119107
ACS	SNOMEDCT_US	15960661000119107
ACS	SNOMEDCT_US	15962541000119106
ACS	SNOMEDCT_US	15963181000119104
ACS	SNOMEDCT_US	15990001
ACS	SNOMEDCT_US	164865005
ACS	SNOMEDCT_US	16837681000119104
ACS	SNOMEDCT_US	17531000119105
ACS	SNOMEDCT_US	194796000
ACS	SNOMEDCT_US	194797009
ACS	SNOMEDCT_US	194799007
ACS	SNOMEDCT_US	194800006
ACS	SNOMEDCT_US	194801005
ACS	SNOMEDCT_US	194802003
ACS	SNOMEDCT_US	194804002
ACS	SNOMEDCT_US	194808004
ACS	SNOMEDCT_US	194810002
ACS	SNOMEDCT_US	194811003

ACS	SNOMEDCT_US	194814006
ACS	SNOMEDCT_US	194816008
ACS	SNOMEDCT_US	194856005
ACS	SNOMEDCT_US	194857001
ACS	SNOMEDCT_US	194858006
ACS	SNOMEDCT_US	194859003
ACS	SNOMEDCT_US	195547003
ACS	SNOMEDCT_US	22298006
ACS	SNOMEDCT_US	23311000119105
ACS	SNOMEDCT_US	233820004
ACS	SNOMEDCT_US	233835003
ACS	SNOMEDCT_US	233838001
ACS	SNOMEDCT_US	233843008
ACS	SNOMEDCT_US	266288001
ACS	SNOMEDCT_US	282006
ACS	SNOMEDCT_US	285721000119104
ACS	SNOMEDCT_US	285981000119103
ACS	SNOMEDCT_US	285991000119100
ACS	SNOMEDCT_US	30277009
ACS	SNOMEDCT_US	304914007
ACS	SNOMEDCT_US	311792005
ACS	SNOMEDCT_US	371068009
ACS	SNOMEDCT_US	394659003
ACS	SNOMEDCT_US	394710008
ACS	SNOMEDCT_US	401303003
ACS	SNOMEDCT_US	401314000
ACS	SNOMEDCT_US	428196007
ACS	SNOMEDCT_US	429731003
ACS	SNOMEDCT_US	4557003

ACS	SNOMEDCT_US	52035003
ACS	SNOMEDCT_US	54329005
ACS	SNOMEDCT_US	57054005
ACS	SNOMEDCT_US	58612006
ACS	SNOMEDCT_US	59063002
ACS	SNOMEDCT_US	62695002
ACS	SNOMEDCT_US	65547006
ACS	SNOMEDCT_US	70211005
ACS	SNOMEDCT_US	703164000
ACS	SNOMEDCT_US	703165004
ACS	SNOMEDCT_US	703209002
ACS	SNOMEDCT_US	703210007
ACS	SNOMEDCT_US	703211006
ACS	SNOMEDCT_US	703212004
ACS	SNOMEDCT_US	703213009
ACS	SNOMEDCT_US	703251009
ACS	SNOMEDCT_US	703252002
ACS	SNOMEDCT_US	703253007
ACS	SNOMEDCT_US	703360004
ACS	SNOMEDCT_US	70998009
ACS	SNOMEDCT_US	72977004
ACS	SNOMEDCT_US	7326005
ACS	SNOMEDCT_US	73795002
ACS	SNOMEDCT_US	73999000
ACS	SNOMEDCT_US	76593002
ACS	SNOMEDCT_US	79009004
ACS	SNOMEDCT_US	91335003
CVA	ICD10CM	I60.00
CVA	ICD10CM	I60.01

CVA	ICD10CM	I60.02
CVA	ICD10CM	I60.1
CVA	ICD10CM	I60.10
CVA	ICD10CM	I60.11
CVA	ICD10CM	I60.12
CVA	ICD10CM	I60.2
CVA	ICD10CM	I60.3
CVA	ICD10CM	I60.30
CVA	ICD10CM	I60.31
CVA	ICD10CM	I60.32
CVA	ICD10CM	I60.4
CVA	ICD10CM	I60.5
CVA	ICD10CM	I60.50
CVA	ICD10CM	I60.51
CVA	ICD10CM	I60.52
CVA	ICD10CM	I60.6
CVA	ICD10CM	I60.7
CVA	ICD10CM	I60.8
CVA	ICD10CM	I60.9
CVA	ICD10CM	I61
CVA	ICD10CM	I61.0
CVA	ICD10CM	I61.1
CVA	ICD10CM	I61.2
CVA	ICD10CM	I61.3
CVA	ICD10CM	I61.4
CVA	ICD10CM	I61.5
CVA	ICD10CM	I61.6
CVA	ICD10CM	I61.8
CVA	ICD10CM	I61.9

CVA	ICD10CM	I63
CVA	ICD10CM	I63.0
CVA	ICD10CM	I63.00
CVA	ICD10CM	I63.01
CVA	ICD10CM	I63.011
CVA	ICD10CM	I63.012
CVA	ICD10CM	I63.013
CVA	ICD10CM	I63.019
CVA	ICD10CM	I63.02
CVA	ICD10CM	I63.03
CVA	ICD10CM	I63.031
CVA	ICD10CM	I63.032
CVA	ICD10CM	I63.033
CVA	ICD10CM	I63.039
CVA	ICD10CM	I63.09
CVA	ICD10CM	I63.1
CVA	ICD10CM	I63.10
CVA	ICD10CM	I63.111
CVA	ICD10CM	I63.112
CVA	ICD10CM	I63.113
CVA	ICD10CM	I63.119
CVA	ICD10CM	I63.12
CVA	ICD10CM	I63.13
CVA	ICD10CM	I63.131
CVA	ICD10CM	I63.132
CVA	ICD10CM	I63.133
CVA	ICD10CM	I63.139
CVA	ICD10CM	I63.19
CVA	ICD10CM	I63.2

CVA	ICD10CM	I63.20
CVA	ICD10CM	I63.21
CVA	ICD10CM	I63.211
CVA	ICD10CM	I63.212
CVA	ICD10CM	I63.213
CVA	ICD10CM	I63.219
CVA	ICD10CM	I63.22
CVA	ICD10CM	I63.23
CVA	ICD10CM	I63.231
CVA	ICD10CM	I63.232
CVA	ICD10CM	I63.233
CVA	ICD10CM	I63.239
CVA	ICD10CM	I63.29
CVA	ICD10CM	I63.3
CVA	ICD10CM	I63.30
CVA	ICD10CM	I63.31
CVA	ICD10CM	I63.311
CVA	ICD10CM	I63.312
CVA	ICD10CM	I63.313
CVA	ICD10CM	I63.319
CVA	ICD10CM	I63.32
CVA	ICD10CM	I63.321
CVA	ICD10CM	I63.322
CVA	ICD10CM	I63.323
CVA	ICD10CM	I63.329
CVA	ICD10CM	I63.33
CVA	ICD10CM	I63.331
CVA	ICD10CM	I63.332
CVA	ICD10CM	I63.333

CVA	ICD10CM	I63.339
CVA	ICD10CM	I63.34
CVA	ICD10CM	I63.341
CVA	ICD10CM	I63.342
CVA	ICD10CM	I63.343
CVA	ICD10CM	I63.349
CVA	ICD10CM	I63.39
CVA	ICD10CM	I63.4
CVA	ICD10CM	I63.40
CVA	ICD10CM	I63.41
CVA	ICD10CM	I63.411
CVA	ICD10CM	I63.412
CVA	ICD10CM	I63.413
CVA	ICD10CM	I63.419
CVA	ICD10CM	I63.42
CVA	ICD10CM	I63.421
CVA	ICD10CM	I63.422
CVA	ICD10CM	I63.423
CVA	ICD10CM	I63.429
CVA	ICD10CM	I63.43
CVA	ICD10CM	I63.431
CVA	ICD10CM	I63.432
CVA	ICD10CM	I63.433
CVA	ICD10CM	I63.439
CVA	ICD10CM	I63.44
CVA	ICD10CM	I63.441
CVA	ICD10CM	I63.442
CVA	ICD10CM	I63.443
CVA	ICD10CM	I63.449

CVA	ICD10CM	I63.49
CVA	ICD10CM	I63.5
CVA	ICD10CM	I63.50
CVA	ICD10CM	I63.51
CVA	ICD10CM	I63.511
CVA	ICD10CM	I63.512
CVA	ICD10CM	I63.513
CVA	ICD10CM	I63.519
CVA	ICD10CM	I63.52
CVA	ICD10CM	I63.521
CVA	ICD10CM	I63.522
CVA	ICD10CM	I63.523
CVA	ICD10CM	I63.529
CVA	ICD10CM	I63.53
CVA	ICD10CM	I63.531
CVA	ICD10CM	I63.532
CVA	ICD10CM	I63.533
CVA	ICD10CM	I63.539
CVA	ICD10CM	I63.54
CVA	ICD10CM	I63.541
CVA	ICD10CM	I63.542
CVA	ICD10CM	I63.543
CVA	ICD10CM	I63.549
CVA	ICD10CM	I63.59
CVA	ICD10CM	I63.6
CVA	ICD10CM	I63.8
CVA	ICD10CM	I63.81
CVA	ICD10CM	I63.89
CVA	ICD10CM	I63.9

CVA	ICD9CM	430
CVA	ICD9CM	431
CVA	ICD9CM	432
CVA	ICD9CM	432.0
CVA	ICD9CM	432.1
CVA	ICD9CM	432.9
CVA	ICD9CM	433
CVA	ICD9CM	433.0
CVA	ICD9CM	433.00
CVA	ICD9CM	433.01
CVA	ICD9CM	433.1
CVA	ICD9CM	433.11
CVA	ICD9CM	433.2
CVA	ICD9CM	433.20
CVA	ICD9CM	433.21
CVA	ICD9CM	433.3
CVA	ICD9CM	433.31
CVA	ICD9CM	433.8
CVA	ICD9CM	433.80
CVA	ICD9CM	433.81
CVA	ICD9CM	433.9
CVA	ICD9CM	433.90
CVA	ICD9CM	433.91
CVA	ICD9CM	434
CVA	ICD9CM	434.0
CVA	ICD9CM	434.00
CVA	ICD9CM	434.01
CVA	ICD9CM	434.1
CVA	ICD9CM	434.10

CVA	ICD9CM	434.11
CVA	ICD9CM	434.9
CVA	ICD9CM	434.90
CVA	ICD9CM	434.91
CVA	ICD9CM	435
CVA	ICD9CM	435.0
CVA	ICD9CM	435.1
CVA	ICD9CM	435.2
CVA	ICD9CM	435.3
CVA	ICD9CM	435.8
CVA	ICD9CM	435.9
CVA	ICD9CM	436
CVA	SNOMEDCT_US	111297002
CVA	SNOMEDCT_US	116288000
CVA	SNOMEDCT_US	140911000119109
CVA	SNOMEDCT_US	140921000119102
CVA	SNOMEDCT_US	14309005
CVA	SNOMEDCT_US	155388006
CVA	SNOMEDCT_US	16000471000119107
CVA	SNOMEDCT_US	16000551000119102
CVA	SNOMEDCT_US	16002031000119102
CVA	SNOMEDCT_US	16002111000119106
CVA	SNOMEDCT_US	16002151000119107
CVA	SNOMEDCT_US	16002511000119104
CVA	SNOMEDCT_US	16023911000119108
CVA	SNOMEDCT_US	16023991000119104
CVA	SNOMEDCT_US	16024031000119100
CVA	SNOMEDCT_US	16024111000119109
CVA	SNOMEDCT_US	16024151000119105

CVA	SNOMEDCT_US	16024191000119100
CVA	SNOMEDCT_US	16024231000119109
CVA	SNOMEDCT_US	16024271000119107
CVA	SNOMEDCT_US	16026951000119102
CVA	SNOMEDCT_US	16026991000119107
CVA	SNOMEDCT_US	16371781000119100
CVA	SNOMEDCT_US	16661971000119104
CVA	SNOMEDCT_US	16891111000119104
CVA	SNOMEDCT_US	195163003
CVA	SNOMEDCT_US	195208004
CVA	SNOMEDCT_US	195212005
CVA	SNOMEDCT_US	195213000
CVA	SNOMEDCT_US	195216008
CVA	SNOMEDCT_US	195217004
CVA	SNOMEDCT_US	230690007
CVA	SNOMEDCT_US	230691006
CVA	SNOMEDCT_US	230698000
CVA	SNOMEDCT_US	230713003
CVA	SNOMEDCT_US	230714009
CVA	SNOMEDCT_US	230715005
CVA	SNOMEDCT_US	25133001
CVA	SNOMEDCT_US	266257000
CVA	SNOMEDCT_US	266312006
CVA	SNOMEDCT_US	270883006
CVA	SNOMEDCT_US	275434003
CVA	SNOMEDCT_US	276222004
CVA	SNOMEDCT_US	277381004
CVA	SNOMEDCT_US	281240008
CVA	SNOMEDCT_US	287731003

CVA	SNOMEDCT_US	292661000119105
CVA	SNOMEDCT_US	292671000119104
CVA	SNOMEDCT_US	292681000119101
CVA	SNOMEDCT_US	292691000119103
CVA	SNOMEDCT_US	313267000
CVA	SNOMEDCT_US	329621000119105
CVA	SNOMEDCT_US	329641000119104
CVA	SNOMEDCT_US	371040005
CVA	SNOMEDCT_US	371041009
CVA	SNOMEDCT_US	371121002
CVA	SNOMEDCT_US	373606000
CVA	SNOMEDCT_US	39925003
CVA	SNOMEDCT_US	413102000
CVA	SNOMEDCT_US	413758000
CVA	SNOMEDCT_US	422504002
CVA	SNOMEDCT_US	426814001
CVA	SNOMEDCT_US	432504007
CVA	SNOMEDCT_US	434141000124103
CVA	SNOMEDCT_US	457551000124104
CVA	SNOMEDCT_US	5471000124102
CVA	SNOMEDCT_US	5571000124103
CVA	SNOMEDCT_US	57357009
CVA	SNOMEDCT_US	62914000
CVA	SNOMEDCT_US	710575003
CVA	SNOMEDCT_US	716051003
CVA	SNOMEDCT_US	723082006
CVA	SNOMEDCT_US	724424009
CVA	SNOMEDCT_US	724425005
CVA	SNOMEDCT_US	724426006

CVA	SNOMEDCT_US	724429004
CVA	SNOMEDCT_US	724993002
CVA	SNOMEDCT_US	724994008
CVA	SNOMEDCT_US	725132001
CVA	SNOMEDCT_US	788880006
CVA	SNOMEDCT_US	788881005
CVA	SNOMEDCT_US	788882003
CVA	SNOMEDCT_US	788883008
CVA	SNOMEDCT_US	788884002
CVA	SNOMEDCT_US	9901000119100
CVA	SNOMEDCT_US	99451000119105

Supplementary Table 2. Historical medical codes used for ACS and CVA event classification.

**Name**	**System**	**Code**
history of ACS	ICD10CM	I23
history of ACS	ICD10CM	I23.0
history of ACS	ICD10CM	I23.1
history of ACS	ICD10CM	I23.2
history of ACS	ICD10CM	I23.3
history of ACS	ICD10CM	I23.4
history of ACS	ICD10CM	I23.5
history of ACS	ICD10CM	I23.6
history of ACS	ICD10CM	I23.7
history of ACS	ICD10CM	I23.8
history of ACS	ICD10CM	I25.2
history of ACS	ICD9CM	412
history of ACS	ICD9CM	429.7
history of ACS	SNOMEDCT_US	428752002
history of ACS	SNOMEDCT_US	138774004

history of ACS	SNOMEDCT_US	138775003
history of ACS	SNOMEDCT_US	155306008
history of ACS	SNOMEDCT_US	161516005
history of ACS	SNOMEDCT_US	164867002
history of ACS	SNOMEDCT_US	1755008
history of ACS	SNOMEDCT_US	233839009
history of ACS	SNOMEDCT_US	233840006
history of ACS	SNOMEDCT_US	233841005
history of ACS	SNOMEDCT_US	233842003
history of ACS	SNOMEDCT_US	308065005
history of ACS	SNOMEDCT_US	32574007
history of ACS	SNOMEDCT_US	399211009
history of ACS	SNOMEDCT_US	698593009
history of CVA	ICD10CM	I69.30
history of CVA	ICD10CM	I69.311
history of CVA	ICD10CM	I69.320
history of CVA	ICD10CM	I69.359
history of CVA	ICD10CM	I69.398
history of CVA	ICD10CM	Z86.73
history of CVA	ICD9CM	438
history of CVA	ICD9CM	438.0
history of CVA	ICD9CM	438.1
history of CVA	ICD9CM	438.10
history of CVA	ICD9CM	438.11
history of CVA	ICD9CM	438.12
history of CVA	ICD9CM	438.13
history of CVA	ICD9CM	438.14
history of CVA	ICD9CM	438.19
history of CVA	ICD9CM	438.2

history of CVA	ICD9CM	438.20
history of CVA	ICD9CM	438.22
history of CVA	ICD9CM	438.3
history of CVA	ICD9CM	438.31
history of CVA	ICD9CM	438.4
history of CVA	ICD9CM	438.40
history of CVA	ICD9CM	438.41
history of CVA	ICD9CM	438.42
history of CVA	ICD9CM	438.5
history of CVA	ICD9CM	438.50
history of CVA	ICD9CM	438.51
history of CVA	ICD9CM	438.52
history of CVA	ICD9CM	438.53
history of CVA	ICD9CM	438.6
history of CVA	ICD9CM	438.7
history of CVA	ICD9CM	438.8
history of CVA	ICD9CM	438.81
history of CVA	ICD9CM	438.82
history of CVA	ICD9CM	438.83
history of CVA	ICD9CM	438.84
history of CVA	ICD9CM	438.89
history of CVA	ICD9CM	438.9
history of CVA	SNOMEDCT_US	118961000119101
history of CVA	SNOMEDCT_US	137991000119103
history of CVA	SNOMEDCT_US	138778001
history of CVA	SNOMEDCT_US	138787005
history of CVA	SNOMEDCT_US	140701000119108
history of CVA	SNOMEDCT_US	140711000119106
history of CVA	SNOMEDCT_US	161506002

history of CVA	SNOMEDCT_US	161511000
history of CVA	SNOMEDCT_US	161518006
history of CVA	SNOMEDCT_US	141281000119101
history of CVA	SNOMEDCT_US	141811000119106
history of CVA	SNOMEDCT_US	141821000119104
history of CVA	SNOMEDCT_US	16896851000119101
history of CVA	SNOMEDCT_US	16896891000119106
history of CVA	SNOMEDCT_US	275526006
history of CVA	SNOMEDCT_US	275527002
history of CVA	SNOMEDCT_US	271395009
history of CVA	SNOMEDCT_US	308067002
history of CVA	SNOMEDCT_US	429235008
history of CVA	SNOMEDCT_US	429993008
history of CVA	SNOMEDCT_US	432051000124108
history of CVA	SNOMEDCT_US	432191000124101
history of CVA	SNOMEDCT_US	440140008
history of CVA	SNOMEDCT_US	690051000119100

Supplementary Table 3. Model features.

**Features**	**Table**	**Lookback (Y)**	**Aggregation**
gender_f	Demographics	100	N/A
gender_m	Demographics	100	N/A
bin_age_0-50	Demographics	100	N/A
bin_age_50-70	Demographics	100	N/A
bin_age_70-inf	Demographics	100	N/A
age	Demographics	100	Any
per_cap_income	Demographics	100	N/A
median_house_income	Demographics	100	N/A
median_family_income	Demographics	100	N/A

height	Demographics	100	Median
family_member_history-family_angina	FH	100	Any
family_member_history-family_arrhythmia	FH	100	Any
family_member_history-family_atherosclerosis	FH	100	Any
family_member_history-family_cad	FH	100	Any
family_member_history-family_cholesterol	FH	100	Any
family_member_history-family_diabetes	FH	100	Any
family_member_history-family_hf	FH	100	Any
family_member_history-family_hypertension	FH	100	Any
family_member_history-family_stroke	FH	100	Any
alcohol-alcohol_status	Alcohol	100	Any
smoke_ever_smoke	Smoking	100	Any
observation-ALP (alkaline phosphatase)_U/L	Observations	10	EWA
observation-ALP (alkaline phosphatase)_U/dL	Observations	10	EWA
observation-ALT_U/L	Observations	10	EWA
observation-ALT_U/dL	Observations	10	EWA
observation-AST_U/L	Observations	10	EWA
observation-AST_U/dL	Observations	10	EWA
observation-BSA_m2	Observations	10	EWA
observation-BUN/Creatinine ratio_mg/dL	Observations	10	EWA
observation-BUN/Creatinine ratio_mg/mg	Observations	10	EWA
observation-BUN/Creatinine ratio_{ratio}	Observations	10	EWA
observation-BUN_mg/dL	Observations	10	EWA
observation-CRP_mg/L	Observations	10	EWA
observation-CRP_mg/dL	Observations	10	EWA
observation-Calcium_mg/dL	Observations	10	EWA
observation-HbA1c_%	Observations	10	EWA
observation-INR_{ratio}	Observations	10	EWA
observation-LDL/HDL ratio_{ratio}	Observations	10	EWA
observation-NRBC_%	Observations	10	EWA
observation-NRBC_/100{WBC}	Observations	10	EWA

observation-NRBC_10*3/uL	Observations	10	EWA
observation-QRS duration_ms	Observations	10	EWA
observation-QT interval_ms	Observations	10	EWA
observation-RBC in serum_/_HPF_	Observations	10	EWA
observation-RBC in serum_/uL	Observations	10	EWA
observation-RBC in serum_10*3/uL	Observations	10	EWA
observation-RBC in serum_10*6/uL	Observations	10	EWA
observation-RBC in serum_U/L	Observations	10	EWA
observation-RBC in urine_/_HPF_	Observations	10	EWA
observation-RBC in urine__HPF_	Observations	10	EWA
observation-RDW cv_%	Observations	10	EWA
observation-RDW sd_fL	Observations	10	EWA
observation-RDW_%	Observations	10	EWA
observation-RDW_fL	Observations	10	EWA
observation-VLDL calc_mg/dL	Observations	10	EWA
observation-VLDL_mg/dL	Observations	10	EWA
observation-WBC in urine_/_HPF_	Observations	10	EWA
observation-WBC in urine__HPF_	Observations	10	EWA
observation-aPTT_s	Observations	10	EWA
observation-albumin (serum)_g/dL	Observations	10	EWA
observation-albumin (urine)_mg/dL	Observations	10	EWA
observation-albumin (urine)_ug/mL	Observations	10	EWA
observation-albumin/creatinine ratio_mg/g	Observations	10	EWA
observation-albumin/globulin ratio_g/dL	Observations	10	EWA
observation-albumin/globulin ratio_mg/dL	Observations	10	EWA
observation-albumin/globulin ratio_mmol/L	Observations	10	EWA
observation-albumin/globulin ratio_{ratio}	Observations	10	EWA
observation-amylase_U/L	Observations	10	EWA
observation-anion gap_mL	Observations	10	EWA
observation-anion gap_mmol/L	Observations	10	EWA
observation-atrial rate_ms	Observations	10	EWA
observation-basophil #_%	Observations	10	EWA

observation-basophil #_/nL	Observations	10	EWA
observation-basophil #_/uL	Observations	10	EWA
observation-basophil #_10*3/uL	Observations	10	EWA
observation-basophil %_%	Observations	10	EWA
observation-bilirubin direct_mg/dL	Observations	10	EWA
observation-bilirubin indirect_mg/dL	Observations	10	EWA
observation-bilirubin total_mg/dL	Observations	10	EWA
observation-bilirubin urine_mg/dL	Observations	10	EWA
observation-carbon dioxide_mmol/L	Observations	10	EWA
observation-casts, urine_/_LPF_	Observations	10	EWA
observation-chloride_mmol/L	Observations	10	EWA
observation-chol/hdl ratio_%	Observations	10	EWA
observation-chol/hdl ratio_mg/dL	Observations	10	EWA
observation-chol/hdl ratio_{ratio}	Observations	10	EWA
observation-creatinine kinase (CK)_U/L	Observations	10	EWA
observation-creatinine, serum_mg/dL	Observations	10	EWA
observation-creatinine, urine_mg/dL	Observations	10	EWA
observation-d-dimer_mg/L	Observations	10	EWA
observation-d-dimer_ng/mL	Observations	10	EWA
observation-d-dimer_ug/mL	Observations	10	EWA
observation-eosinophils #_%	Observations	10	EWA
observation-eosinophils #_/uL	Observations	10	EWA
observation-eosinophils #_10*3/uL	Observations	10	EWA
observation-eosinophils %_%	Observations	10	EWA
observation-epithelial cells_/_HPF_	Observations	10	EWA
observation-estimated average glucose (eAG)_mg/dL	Observations	10	EWA
observation-estimated average glucose (eAG)_mmol/L	Observations	10	EWA
observation-estradiol_pg/mL	Observations	10	EWA
observation-ferritin_ng/mL	Observations	10	EWA
observation-ferritin_ug/L	Observations	10	EWA
observation-folate_ng/mL	Observations	10	EWA

observation-fsh_uU/mL	Observations	10	EWA
observation-globulin_g/dL	Observations	10	EWA
observation-glucose, serum_mg/dL	Observations	10	EWA
observation-glucose, urine_mg/dL	Observations	10	EWA
observation-hct_%	Observations	10	EWA
observation-height__in_i_	Observations	10	EWA
observation-height_cm	Observations	10	EWA
observation-hemoglobin_g/dL	Observations	10	EWA
observation-immature granulocytes #_%	Observations	10	EWA
observation-immature granulocytes #_10*3/uL	Observations	10	EWA
observation-immature granulocytes %_%	Observations	10	EWA
observation-insulin_uU/mL	Observations	10	EWA
observation-iron binding capacity_ug/dL	Observations	10	EWA
observation-iron saturation_%	Observations	10	EWA
observation-iron_ug/dL	Observations	10	EWA
observation-ketones, urine_mg/dL	Observations	10	EWA
observation-leukocytes (WBC)_/_HPF_	Observations	10	EWA
observation-leukocytes (WBC)_/nL	Observations	10	EWA
observation-leukocytes (WBC)_/uL	Observations	10	EWA
observation-leukocytes (WBC)_10*3/uL	Observations	10	EWA
observation-lh_uU/mL	Observations	10	EWA
observation-lipase_U/L	Observations	10	EWA
observation-lymphocytes #_%	Observations	10	EWA
observation-lymphocytes #_/uL	Observations	10	EWA
observation-lymphocytes #_10*3/uL	Observations	10	EWA
observation-lymphocytes %_%	Observations	10	EWA
observation-magnesium_mg/dL	Observations	10	EWA
observation-magnesium_mmol/L	Observations	10	EWA
observation-mch_pg	Observations	10	EWA
observation-mch_pg/dL	Observations	10	EWA
observation-mchc_%	Observations	10	EWA

observation-mchc_g/dL	Observations	10	EWA
observation-mcv_fL	Observations	10	EWA
observation-monocytes #_%	Observations	10	EWA
observation-monocytes #_/nL	Observations	10	EWA
observation-monocytes #_/uL	Observations	10	EWA
observation-monocytes #_10*3/uL	Observations	10	EWA
observation-monocytes %_%	Observations	10	EWA
observation-mpv_fL	Observations	10	EWA
observation-neutrophils #_%	Observations	10	EWA
observation-neutrophils #_/nL	Observations	10	EWA
observation-neutrophils #_/uL	Observations	10	EWA
observation-neutrophils #_10*3/uL	Observations	10	EWA
observation-neutrophils %_%	Observations	10	EWA
observation-non-HDL cholesterol_mg/dL	Observations	10	EWA
observation-osmolality calc_mosm/L	Observations	10	EWA
observation-osmolality calc_mosm/kg	Observations	10	EWA
observation-p axis_deg	Observations	10	EWA
observation-p-r interval_ms	Observations	10	EWA
observation-ph, blood_U	Observations	10	EWA
observation-ph, blood__pH_	Observations	10	EWA
observation-ph, urine_U	Observations	10	EWA
observation-ph, urine__pH_	Observations	10	EWA
observation-phosphorus_mg/dL	Observations	10	EWA
observation-platelet_/nL	Observations	10	EWA
observation-platelet_/uL	Observations	10	EWA
observation-platelet_10*3/uL	Observations	10	EWA
observation-potassium_mmol/L	Observations	10	EWA
observation-procalcitonin_ng/mL	Observations	10	EWA
observation-progesterone_ng/mL	Observations	10	EWA
observation-prolactin_ng/mL	Observations	10	EWA
observation-protein total, serum_g/dL	Observations	10	EWA
observation-protein total, serum_mg/dL	Observations	10	EWA

observation-protein, urine_mg/dL	Observations	10	EWA
observation-psa_ng/mL	Observations	10	EWA
observation-pt_s	Observations	10	EWA
observation-ptt_s	Observations	10	EWA
observation-qrs axis_deg	Observations	10	EWA
observation-r axis_deg	Observations	10	EWA
observation-respiratory rate_{Breaths}/min	Observations	10	EWA
observation-rheumatoid factor_U/mL	Observations	10	EWA
observation-sedimentation rate (ESR)_mm/h	Observations	10	EWA
observation-sodium_mmol/L	Observations	10	EWA
observation-spo2 %_%	Observations	10	EWA
observation-t axis_deg	Observations	10	EWA
observation-t3_pg/mL	Observations	10	EWA
observation-t4, free_ng/dL	Observations	10	EWA
observation-temperature oral_Cel	Observations	10	EWA
observation-temperature temporal artery_Cel	Observations	10	EWA
observation-testosterone_ng/dL	Observations	10	EWA
observation-triglyceride_mg/dL	Observations	10	EWA
observation-troponin i_ng/L	Observations	10	EWA
observation-troponin i_ng/mL	Observations	10	EWA
observation-troponin i_pg/mL	Observations	10	EWA
observation-tsh_uU/L	Observations	10	EWA
observation-tsh_uU/mL	Observations	10	EWA
observation-uric acid_mg/dL	Observations	10	EWA
observation-urobilinogen, urine_mg/dL	Observations	10	EWA
observation-vitamin b-12_pg/mL	Observations	10	EWA
observation-vitamin d_ng/mL	Observations	10	EWA
observation-weight__lb_av_	Observations	10	EWA
observation-weight_kg	Observations	10	EWA
observation-egfr_together_mixed	Observations	10	EWA
bp_meds-bp_meds	Medications	100	Any
cholesterol_meds-cholesterol_meds	Medications	100	Any

cholesterol_meds-statin_meds	Medications	100	Any
cholesterol-hdl	Cholesterol	10	Last
cholesterol-ldl	Cholesterol	10	Last
cholesterol-total	Cholesterol	10	Last
cholesterol-triglycerides	Cholesterol	10	Last
chosen_conditions-RTI	Conditions	100	Any
chosen_conditions-atrial_fibrillation	Conditions	100	Any
chosen_conditions-bells_palsy	Conditions	100	Any
chosen_conditions-diabetes_1	Conditions	100	Any
chosen_conditions-diabetes_2	Conditions	100	Any
chosen_conditions-erectile_dysfunction	Conditions	100	Any
chosen_conditions-hypertension	Conditions	100	Any
chosen_conditions-kidney_disease	Conditions	100	Any
chosen_conditions-lupus_erythematosu	Conditions	100	Any
chosen_conditions-mental_illness	Conditions	100	Any
chosen_conditions-migraines	Procedures	100	Any
chosen_conditions-rheumatoid_arthritis	Procedures	100	Any
chosen_procedures-artries_and_veins_surgeries	Procedures	100	Any
chosen_procedures-bnp	Procedures	100	Any
chosen_procedures-cardiac_catheterization_procedures	Procedures	100	Any
chosen_procedures-cardiography_procedures	Procedures	100	Any
chosen_procedures-cardiovascular_monitoring_services	Procedures	100	Any
chosen_procedures-cerebrovascular_arterial_studies	Procedures	100	Any
chosen_procedures-chest_vessels	Procedures	100	Any
chosen_procedures-ck	Procedures	100	Any
chosen_procedures-diagnostic_nuclear_medicine_procedures_on_cardiovascular_system	Procedures	100	Any

chosen_procedures-diagnostic_radiology_procedures_of_the_heart	Procedures	100	Any
chosen_procedures-echocardiography_procedures	Procedures	100	Any
chosen_procedures-extremity_arterial_studies	Procedures	100	Any
chosen_procedures-extremity_venous_studies	Procedures	100	Any
chosen_procedures-other_cardiovascular_procedures	Procedures	100	Any
chosen_procedures-pulmonary_vessels	Procedures	100	Any
chosen_procedures-troponin	Procedures	100	Any
chosen_procedures-visceral_and_penile_vascular_studies	Procedures	100	Any
bps-diastolic_bp	BP	10	Last
bps-heart_rate	BP	10	Last
bps-systolic_bp	BP	10	Last
app_bps-systolic_bp_mixed	BP+mHealth	100	Any
app_bps-diastolic_bp_mixed	BP+mHealth	100	Any
app_bps-heart_rate_mixed	BP+mHealth	100	Any
num_records_app_bps_90	mHealth	0.25	Any
num_records_90	EHR Combined	0.25	N/A
num_records	EHR Combined	100	N/A
emb_CBOW_CO_embeddings_50_0	EHR Combined	100	Mean
emb_CBOW_CO_embeddings_50_1	EHR Combined	100	Mean
emb_CBOW_CO_embeddings_50_2	EHR Combined	100	Mean
emb_CBOW_CO_embeddings_50_3	EHR Combined	100	Mean
emb_CBOW_CO_embeddings_50_4	EHR Combined	100	Mean
emb_CBOW_CO_embeddings_50_5	EHR Combined	100	Mean
emb_CBOW_CO_embeddings_50_6	EHR Combined	100	Mean
emb_CBOW_CO_embeddings_50_7	EHR Combined	100	Mean
emb_CBOW_CO_embeddings_50_8	EHR Combined	100	Mean
emb_CBOW_CO_embeddings_50_9	EHR Combined	100	Mean
emb_CBOW_CO_embeddings_50_10	EHR Combined	100	Mean

emb_CBOW_CO_embeddings_50_11	EHR Combined	100	Mean
emb_CBOW_CO_embeddings_50_12	EHR Combined	100	Mean
emb_CBOW_CO_embeddings_50_13	EHR Combined	100	Mean
emb_CBOW_CO_embeddings_50_14	EHR Combined	100	Mean
emb_CBOW_CO_embeddings_50_15	EHR Combined	100	Mean
emb_CBOW_CO_embeddings_50_16	EHR Combined	100	Mean
emb_CBOW_CO_embeddings_50_17	EHR Combined	100	Mean
emb_CBOW_CO_embeddings_50_18	EHR Combined	100	Mean
emb_CBOW_CO_embeddings_50_19	EHR Combined	100	Mean
emb_CBOW_CO_embeddings_50_20	EHR Combined	100	Mean
emb_CBOW_CO_embeddings_50_21	EHR Combined	100	Mean
emb_CBOW_CO_embeddings_50_22	EHR Combined	100	Mean
emb_CBOW_CO_embeddings_50_23	EHR Combined	100	Mean
emb_CBOW_CO_embeddings_50_24	EHR Combined	100	Mean
emb_CBOW_CO_embeddings_50_25	EHR Combined	100	Mean
emb_CBOW_CO_embeddings_50_26	EHR Combined	100	Mean
emb_CBOW_CO_embeddings_50_27	EHR Combined	100	Mean
emb_CBOW_CO_embeddings_50_28	EHR Combined	100	Mean
emb_CBOW_CO_embeddings_50_29	EHR Combined	100	Mean
emb_CBOW_CO_embeddings_50_30	EHR Combined	100	Mean
emb_CBOW_CO_embeddings_50_31	EHR Combined	100	Mean
emb_CBOW_CO_embeddings_50_32	EHR Combined	100	Mean
emb_CBOW_CO_embeddings_50_33	EHR Combined	100	Mean
emb_CBOW_CO_embeddings_50_34	EHR Combined	100	Mean
emb_CBOW_CO_embeddings_50_35	EHR Combined	100	Mean
emb_CBOW_CO_embeddings_50_36	EHR Combined	100	Mean
emb_CBOW_CO_embeddings_50_37	EHR Combined	100	Mean
emb_CBOW_CO_embeddings_50_38	EHR Combined	100	Mean
emb_CBOW_CO_embeddings_50_39	EHR Combined	100	Mean
emb_CBOW_CO_embeddings_50_40	EHR Combined	100	Mean
emb_CBOW_CO_embeddings_50_41	EHR Combined	100	Mean
emb_CBOW_CO_embeddings_50_42	EHR Combined	100	Mean

emb_CBOW_CO_embeddings_50_43	EHR Combined	100	Mean
emb_CBOW_CO_embeddings_50_44	EHR Combined	100	Mean
emb_CBOW_CO_embeddings_50_45	EHR Combined	100	Mean
emb_CBOW_CO_embeddings_50_46	EHR Combined	100	Mean
emb_CBOW_CO_embeddings_50_47	EHR Combined	100	Mean
emb_CBOW_CO_embeddings_50_48	EHR Combined	100	Mean
emb_CBOW_CO_embeddings_50_49	EHR Combined	100	Mean

Supplementary Table 4. Lookback period and aggregation.

**Resource type**	**Lookback (Y)**	**Aggregation method**
Family history	100	Any
Smoking	100	Any
Alcohol	100	Any
Observations	10	EWA
Medication	100	Any
Cholesterol	10	Last
Blood pressure	10	Last
Conditions	100	Any
Procedures	100	Any
ICD10 embedding	100	Mean

2.2 Supplementary Figures

Supplementary Figure 1. ACS labeling protocol and counts.

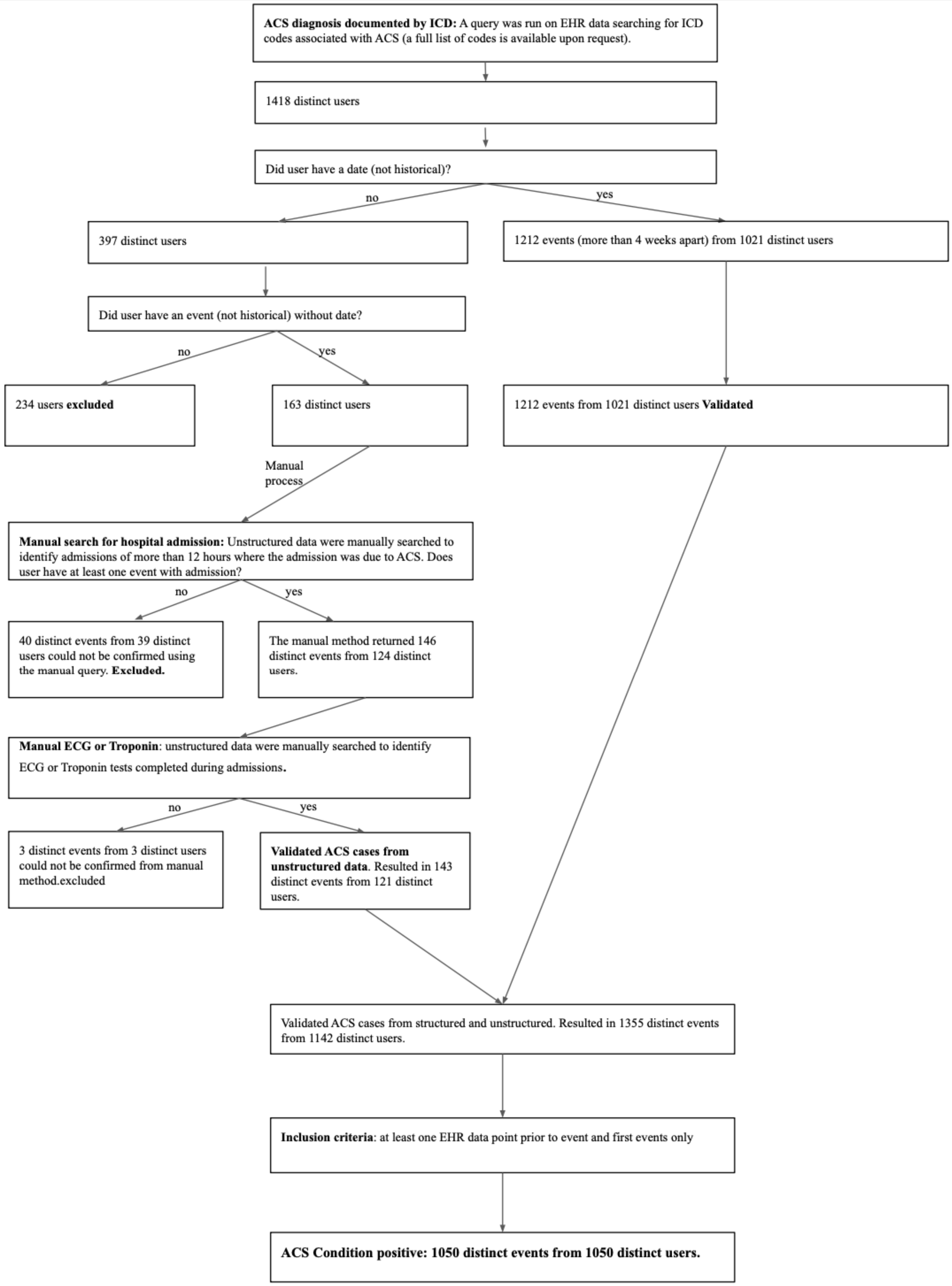

Supplementary Figure 2. CVA labeling protocol and counts.

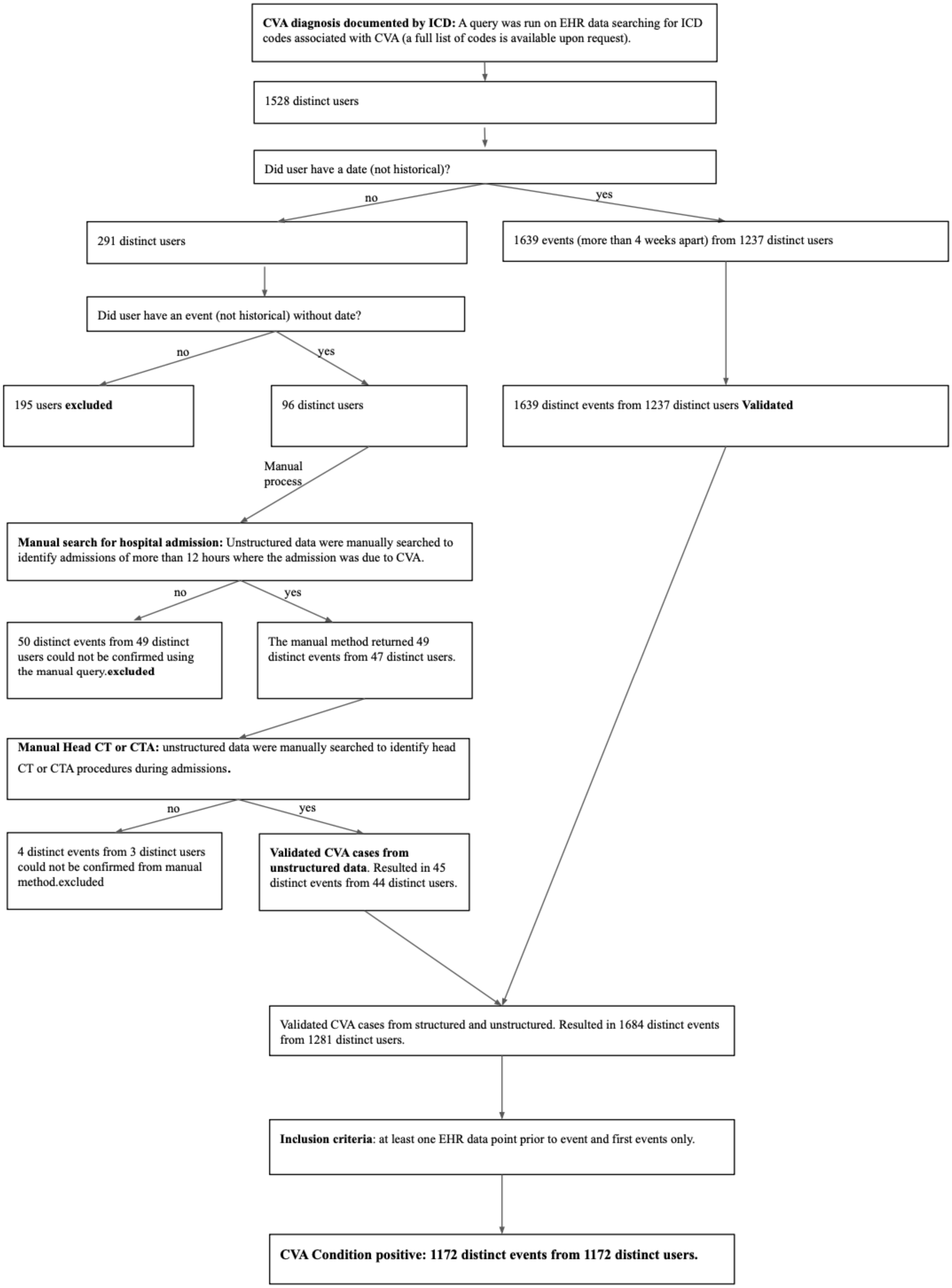

Supplementary Figure 3. Percent positive for high vs. low model prediction (prediction values).

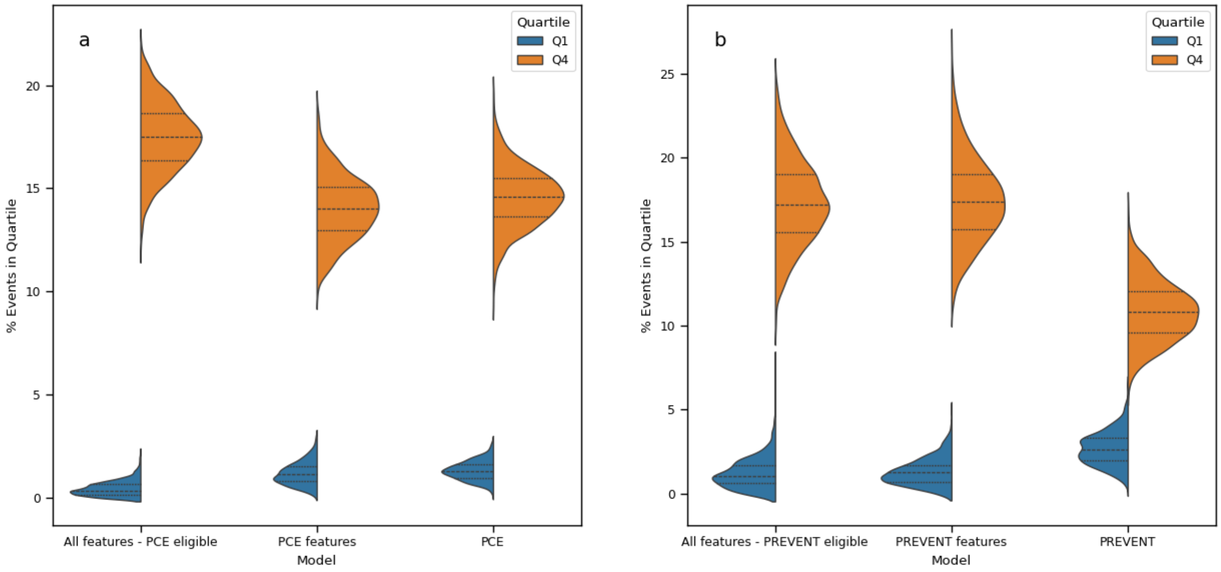

4th quantile (25% with the highest prediction scores) in orange vs 1st quantile (25% with lowest prediction scores) in blue. Distributions are calculated from the 1000 bootstrapping iterations. (a) PCE eligible population (b) PREVENT eligible population.

Supplementary Figure 4. Shapley values beeswarm plots (a) 90-day prediction (b) 365-day prediction.

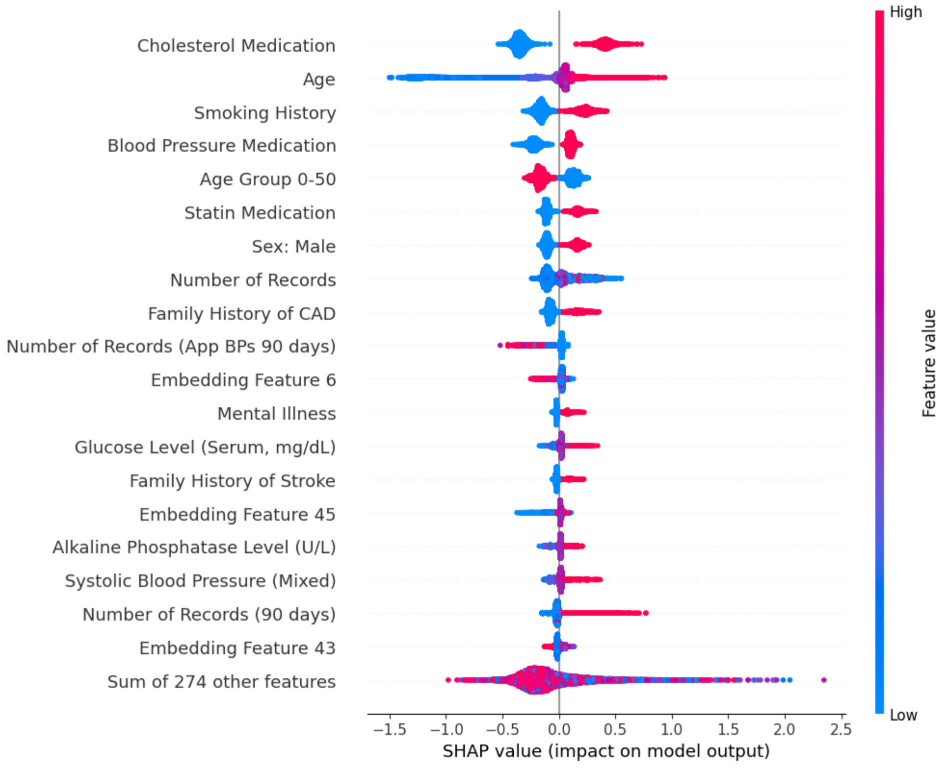

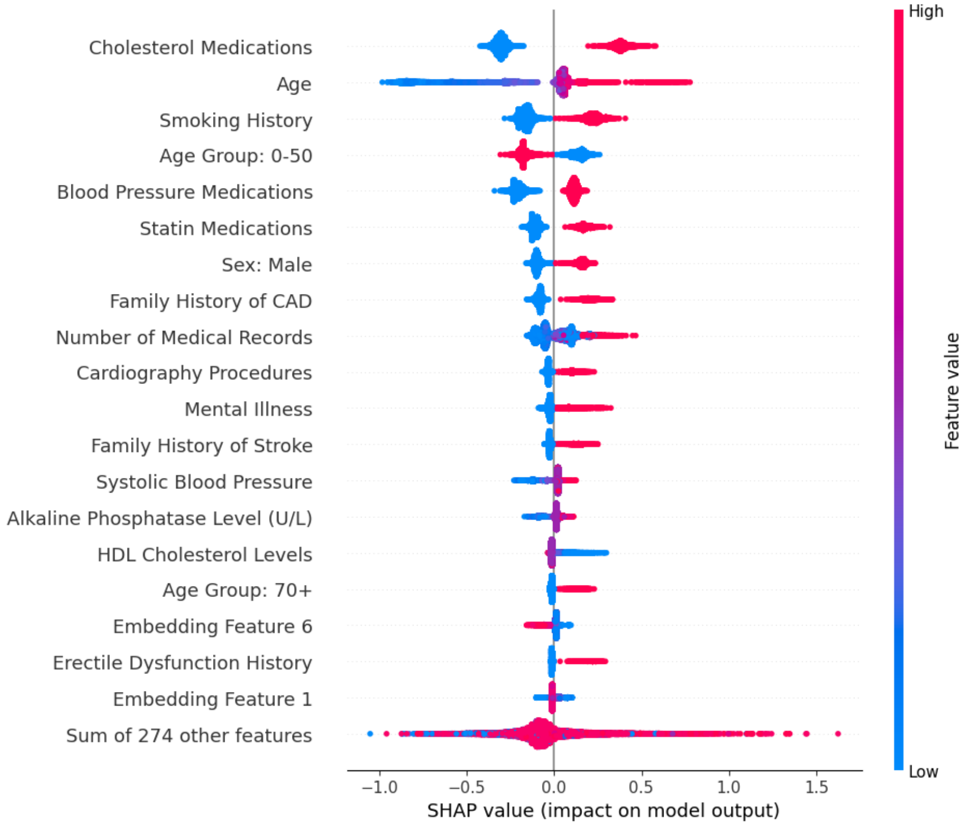

Supplementary Figure 5. ROC plots 90-day (left) and 365-day (right) prediction periods for the population with mHealth data before ASCVD event.

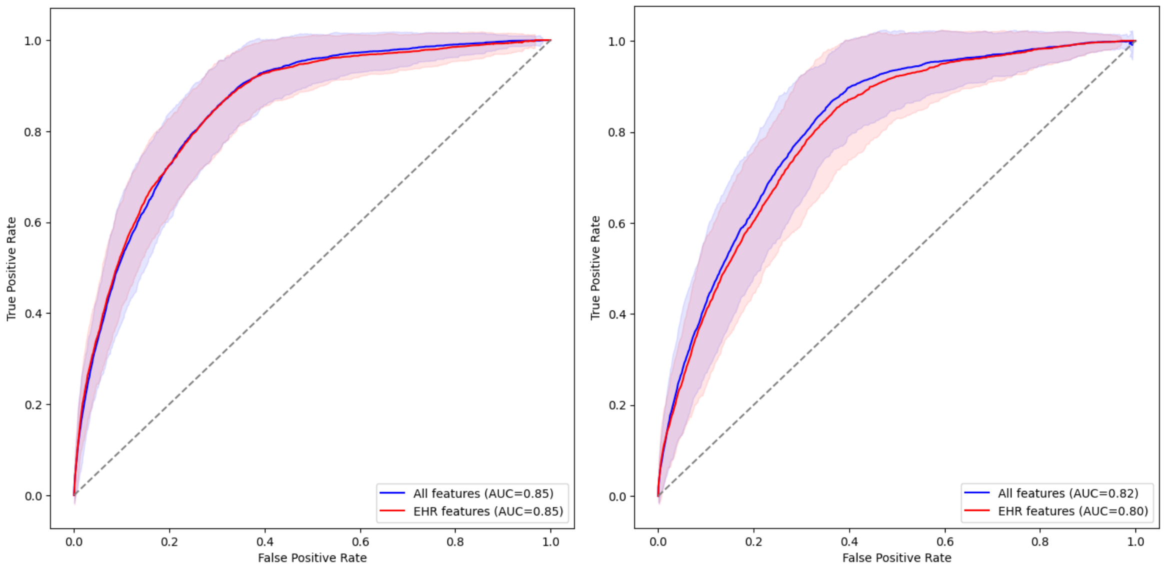

Supplementary Figure 6. Shapley values beeswarm plots 90-day (left) and 365-day (right) prediction periods for the population with mHealth data before ASCVD event.

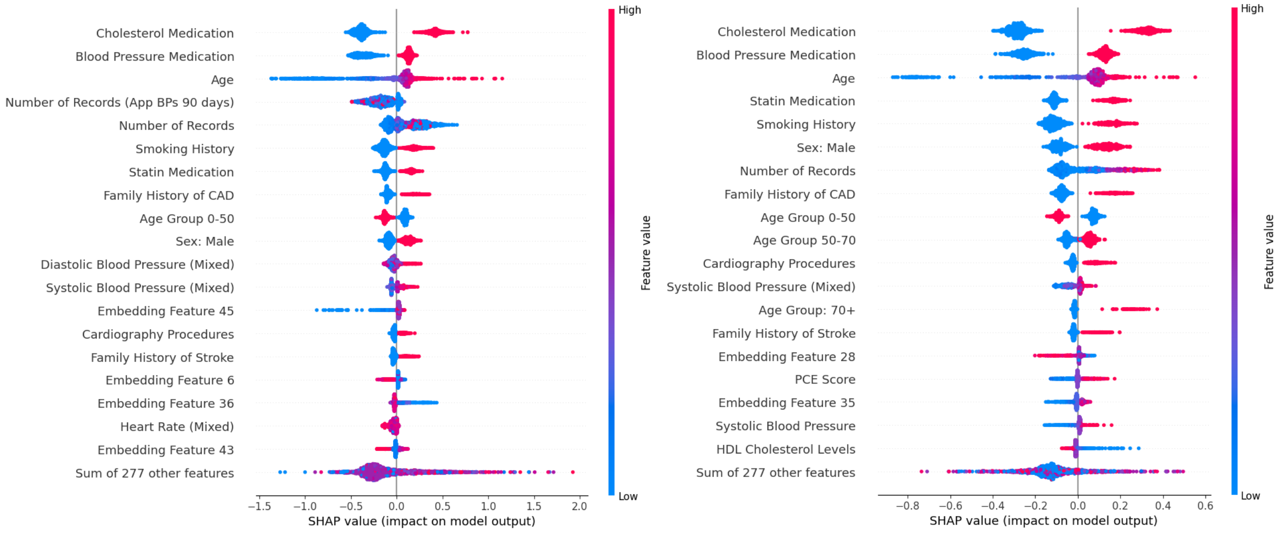

Supplement: Supplementary file 1 [file Datasheet1.pdf]
